# Nutrient Intake and Status of German Children and Adolescents Consuming Vegetarian, Vegan or Omnivore Diets: Results of the VeChi Youth Study

**DOI:** 10.3390/nu13051707

**Published:** 2021-05-18

**Authors:** Ute Alexy, Morwenna Fischer, Stine Weder, Alfred Längler, Andreas Michalsen, Andreas Sputtek, Markus Keller

**Affiliations:** 1 Department of Nutritional Epidemiology, Institute of Nutritional and Food Science, University of Bonn, 44225 Dortmund, Germany; 2 Faculty of Human Resources, Health & Social Work, University of Applied Sciences (FHM), 33602 Bielefeld, Germany; morwenna.hoffmann@fh-mittelstand.de; 3 Research Institute of Plant-Based Nutrition, 35444 Gießen/Biebertal, Germany; weder@ifpe-giessen.de (S.W.); keller@ifpe-giessen.de (M.K.); 4 Faculty of Health, Gemeinschaftskrankenhaus Herdecke, Witten Herdecke University, 58313 Herdecke, Germany; a.laengler@gemeinschaftskrankenhaus.de; 5 Institute of Social Medicine, Epidemiology and Health Economics, Charité-Universitätsmedizin Berlin, 10117 Berlin, Germany; andreas.michalsen@immanuelalbertinen.de; 6 Medical Laboratory Bremen GmbH, 28359 Bremen, Germany; andreas.sputtek@mlhb.de

**Keywords:** vegetarian diet, vegan diet, children, adolescents, nutrient status, biomarker, blood lipids, dietary intake

## Abstract

There is a lack of data on associations between modern vegetarian and vegan diets and health among children and adolescents. The aim of the Vechi Youth Study was to cross-sectionally examine anthropometry, dietary intakes and nutritional status in a sample of 149 vegetarian, 115 vegan and 137 omnivore children and adolescents (6–18 years old, mean age: 12.7 ± 3.9 years). Group differences of dietary intake (calculated from three-day dietary records), nutrient biomarker and blood lipid concentrations were assessed using an analysis of covariance, adjusted for sex, age and other covariates. The total energy intake did not differ significantly between groups, but intake of carbohydrates was higher among vegetarians and vegans than among omnivores (*p* = 0.0002, respectively). The median protein intake exceeded 0.9 g/kg body weight/day in all diet groups and was lowest among vegetarians (*p* < 0.02). There was no significant difference of haemoglobin, vitamin B2, 25-OH vitamin D3, HDL-C and triglycerides blood concentrations between diet groups. Vegan participants had higher folate concentrations than vegetarian participants (*p* = 0.0053). Ferritin concentration was significantly higher in omnivores than in vegetarians (*p* = 0.0134) and vegans (*p* = 0.0404). Vegetarians had lower concentrations of holotranscobalamin (*p* = 0.0042) and higher concentrations of methylmalonic acid (*p* = 0.0253) than omnivores. Vegans had the lowest non-HDL-C and LDL-C concentrations in comparison to vegetarians (*p* = 0.0053 and *p* = 0.0041) and omnivores (*p* = 0.0010 and *p* = 0.0010). A high prevalence (>30%) of 25-OH vitamin D3 and vitamin B2 concentrations below reference values were found irrespective of the diet group. In conclusion, the Vechi Youth Study did not indicate specific nutritional risks among vegetarian and vegan children and adolescents compared to omnivores.

## 1. Introduction

Plant-based, i.e., vegetarian (excluding meat and fish) and vegan (excluding all foods from animal origins), diets have become more popular in Western societies during the last decades [1], including in children and adolescents. In the recent German representative KiGGS Study, 3.4% of children and adolescents followed a vegetarian (including vegan) diet in 2015–2017, which was an increase since 2006 [2]. Among adults, a recent survey in 2016 estimated the total number of vegans in Germany at 1.3 million (1.6% of adults) [3].

As each restriction of food group intake in the diet increases the risk of nutritional deficits, there is a controversy as to whether or not vegetarian and in particular vegan diets are appropriate during growth [4,5]. Several nutrients, i.e., protein, vitamin B12, vitamin B2, calcium and selenium are regarded as critical in vegan diets, in addition to iron, zinc and long-chained n-3-fatty acids in vegetarian diets [4,6,7]. Additionally, in an omnivore diet (including both food from animal and plant origins), studies indicate a suboptimal status of vitamin D and iodine in childhood and adolescence [8,9].

However, there is a lack of data on the current practice of vegetarian and vegan diets [10,11], in particular in children and adolescents. A recent review listed a total of 16 studies on this issue among children and adolescents published until 2014, which were mostly cross-sectional, outdated (mainly from the 1970–90s), and with small sample sizes. Only two of these studies evaluated vegan participants as a separate group [10]. To our knowledge, after the publication of the aforementioned review, only a few more studies on vegetarian and vegan diets during growth have been conducted. Three of them were published by a Polish working group examining in a cross-sectional design the vitamin status, inflammation and bone health in vegetarian and/or vegan children [12,13,14]. Another two cross-sectional studies from Scandinavia compared the dietary intake and nutrient status of Swedish vegans and omnivores aged 6–20 years (*n* = 30) [15,16], and of Finnish children (*n* = 40, median age 3.5 years) on a vegan, vegetarian (including fish-eaters) or omnivore diet [17]. A further cross-sectional study, the German VeChi Diet Study, was conducted by our working group and examined dietary intakes of 430 vegetarian, vegan or omnivore toddlers aged one to three years [18].

Hence, there is a need to investigate the current nutritional and health status of children and adolescents on a modern vegetarian or vegan diet. Thus, the main objective of the Vegetarian and Vegan Youth Study (VeChi Youth Study) was to examine nutritional status, dietary intake and anthropometrics among children and adolescents aged 6–18 years in Germany. Here, we present data on the dietary intake and nutrient status of the participants of the VeChi Youth Study.

## 2. Materials and Methods

### 2.1. Study Design

The VeChi Youth Study is a cross-sectional study collecting data on anthropometrics, diet, lifestyle and nutritional status among 401 vegetarian, vegan and omnivore children and adolescents aged 6–18 years between October 2017 and January 2019 in three study centres in Germany. Primary results of the study were published in the German 14th Nutrition Report [19].

The study was conducted according to the guidelines of the Declaration of Helsinki and approved by the ethics committee of the University of Witten-Herdecke (139/2017). The study has been registered at the German Clinical Trials Register (DRKS00012835).

### 2.2. Recruitment

Subjects were recruited mainly via the study website (www.vechi-youth-studie.de, accessed on 17 May 2021), social media groups on plant-based diets, flyers, magazines, journals and vegetarian or vegan societies’ websites from September 2017 until October 2018. Inclusion criteria were as follows: healthy vegetarian, vegan or omnivore children and adolescents (age 6–18 years) living in Germany. Exclusion criteria were as follows: (a) diagnosed diseases that could affect the studied variables (e.g., enteropathy, pancreatic diseases, metabolic disorders like phenylketonuria or fructose malabsorption), and (b) special diets other than vegan or vegetarian diet, e.g., predominantly (≥70%) raw food diet (according to [20]). One participant age 5.5 years and one participant age 19.1 years were also included into the study sample.

### 2.3. Study Schedule

If study inclusion criteria were met, participants received detailed written study information. After the parents of younger (<14 years) children and older participants themselves gave their written consent for study participation, they were invited to one study centre (Department of Integrative Paediatric and Adolescent Medicine, Gemeinschaftskrankenhaus, Herdecke in the West of Germany, Department of Internal and Integrative Medicine, Immanuel Krankenhaus Berlin, in the East of Germany or Filderklinik, Filderstadt-Bonlanden, in the South of Germany). The latter study centre cancelled participation after the examination of eleven participants due to organizational problems. During the visits in the study centres, anthropometric measurements were performed by trained staff and blood and spot urine samples were taken. Participants or parents of younger participants completed questionnaires on physical activity and puberty status. Additionally, participants were asked to provide a 3-day weighed food record at home and to complete an online questionnaire. For study participation, children and adolescents received an incentive of 50 € each and were provided with the results of the dietary records and blood analyses.

### 2.4. Anthropometric Measurements

Body weight (in underwear, without shoes, Seca 799, graduation 100 g, up to 150 kg body weight) and height (stadiometer Seca 222, graduation 1 mm) were measured at the study centres.

### 2.5. Blood Samples

Venous blood samples (<20 mL) were drawn during the study centre visits after an overnight fast. To verify the fasting, participants and/or their parents were asked about the timing of the last eating occasion and the consumption of caloric beverages. All blood parameters were determined at the MVZ Medical Laboratory Bremen GmbH, which is accredited by DAkkS (Deutsche Akkreditierungsstelle, a national accreditation body of the Federal Republic of Germany). Haemoglobin, vitamin B2 (riboflavin) and folate were determined using EDTA whole blood samples stored and transported at 4–8 °C for a maximum of 3 days. Serum samples were obtained by centrifugation at 2500× *g* for 10 min within 1 h after the withdrawal. They were stored frozen below −20 °C for a maximum of 3 months and shipped to the laboratory over dry ice prior to the measurements. Serum samples served for the measurement of ferritin, 25-OH vitamin D3, holotranscobalamin (holoTC), methylmalonic acid (MMA), triglycerides and total, LDL and HDL cholesterol. Haemoglobin determinations took place photometrically as sodiumlaurylsulfate hemoglobin using an automatic analyser (XN-9000, Sysmex, Norderstedt, Germany). For the measurement of vitamin B2 (as flavin adenine dinucleotide, FAD) we applied an isocratic HPLC in-house method with fluorimetric detection. Ferritin and folate were measured by means of electrochemiluminescence immunoassays (Cobas, Roche Diagnostics, Mannheim, Germany). The determination of 25-OH vitamin D3 was done by means of a chemiluminescence immune assay (Diasorin, Dietzenbach, Germany). An enzymatic immunoassay (IBL International, Hamburg, Germany) was applied for the measurement of holoTC, and a gas chromatographic in-house method (not polar capillary separation column with single ion monitoring (SIM) detection) provided the results for the concentrations of methyl malonic acid. Total, HDL, non-HDl and LDL cholesterol as well as triglyceride concentrations were measured using enzymatic colour tests from Roches Diagnostics running on a Cobas automatic analyser. The day-to-day coefficients of the analytical methods varied from 4% to 10% depending on the method.

Blood sampling was not successful in five children. There were some further missing values due to insufficient sample volume or technical problems during the analysis (haemoglobin and folate *n* = 3, ferritin *n* = 1, vitamin B2 *n* = 4, holoTC *n* = 9, MMA *n* = 2). The following reference values were used to assess the respective nutrient and blood lipid status:

- Anaemia: haemoglobin <11.5 g/dL (6–<12 years), <12 g/dL (12–<15 years and girls >15 years), <13 g/dL (boys >15 years) [21]
- Depleted iron stores: ferritin < 12–15 µg/L [22]
- 25-OH Vitamin D3: subnormal 30–50 nmol/L; deficiency <30 nmol/L [9,23]
- Vitamin B2: deficiency FAD < 199 µg/L [24]
- Folate: deficiency < 226.5 µg/L [25]
- Vitamin B12:
  deficiency unlikely: HoloTC > 50 pmol/L or holoTC 35–50 pmol/L and MMA < 271 nmol/L;
  negative vitamin B12 balance: holoTC < 35 pmol/L and MMA < 271 nmol/L;
  deficiency likely: holoTC < 35 pmol/L and MMA > 271 nmol/L [26]
- Blood lipids: acceptable: total cholesterol (TC) <170 mg/dL, HDL-cholesterol (HDL-C) >45 mg/dL, LDL-cholesterol (LDL-C): <110 mg/dL (optimal), triglycerides (TG): <74 mg/dL (6–9 years), <90 mg/dL (10–19 years), non-HDL-cholesterol (non-HDL-C): <123 mg/dL [27].

### 2.6. Dietary Assessment

Dietary intake was assessed using 3-day weighed dietary records as described previously [18,28]. The participating families chose the day of the beginning of dietary recording within a given period. All foods and beverages consumed, as well as leftovers, were weighed and recorded over 3 days using electronic kitchen scales. Packages of commercial food products were also provided. When exact weighing was not possible (e.g., in the case of eating out), semi-quantitative household recording with measures (e.g., spoons, cups) was allowed. Missing data were assessed by the study staff by requesting the information from the parents via email. Energy and nutrient intakes were calculated as individual means of the 3 days of recording using the food composition database LEBTAB [29]. The composition of staple foods was based on the standard German food composition tables (Bundeslebensmittelschlüssel BLS 3.02). LEBTAB was continuously updated by adding those foods or nutrient supplements recorded by study participants. Energy and nutrient contents of commercial food products, i.e., processed foods and ready-to-eat-meals or snack foods, were estimated by recipe simulation using labelled ingredients and nutrient contents including fortified nutrients. Dietary supplements were not considered for nutrient intake calculation in this analysis, as the focus of the evaluation was on nutrient intake from food. Records were considered as underreported when the total energy intake was inadequate in relation to the estimated basal metabolic rate (BMR) (according to age- and sex-specific equations of Schofield [30]. Paediatric cut-offs [31] were used to identify underreported records.

### 2.7. Questionnaires

Sociodemographic variables (e.g., nationality, size of residential town, income per month, parental education and profession and parental body weight and height) and further dietary variables (e.g., duration of exposure to a vegetarian or vegan diet, main motivation for the practice of a vegetarian or vegan diet and use of supplements) were assessed using an online questionnaire. Physical activity was assessed by a questionnaire based on the validated Adolescent Physical Activity Recall Questionnaire [32] including questions on organized (e.g., training in a sports club) and non-organized sport activities (leisure sports, e.g., jogging or playing football with friends). Puberty stages according to Tanner were assessed by a questionnaire including schematic drawings [33,34].

### 2.8. Diet Group Classification

The three diet groups (i.e., vegetarian, vegan or omnivore) were categorized according to the following question during recruitment:

Do you/does your child eat

- a vegetarian diet (no meat, sausage, fish, but dairy and/or eggs)
- a vegan diet (no meat, sausage, fish, dairy and eggs)
- an omnivore diet (including meat and/or fish)?

In addition, in the online questionnaire, some crosscheck questions were asked, whether there are exceptions in food intake, for example vegan children and adolescents consuming cow’s milk or vegetarian children occasionally eating fish or meat. According to these crosscheck questions some participants were re-assigned: vegetarian and vegan children or adolescents who usually eat meat or fish ≥ 1 time/week were reclassified as omnivore (*n* = 6). Vegan subjects who usually eat dairy products and/or eggs ≥ 1 time/week were categorized as vegetarian (*n* = 16).

### 2.9. Statistics

The power calculation was carried out with G-Power for serum ferritin as the primary outcome. A survey of 42 vegetarian and 56 omnivore children from Taiwan aged 2–6 years [35] showed a difference of serum ferritin of 12.1 ng/mL between the two groups. On this basis, a medium effect size of Cohen’s d = 0.6 was anticipated. This resulted in a case number of *n* = 35 per age and diet group with a power of β = 0.8 and a one-sided level α = 0.05 (a priori two-sided *t*-test). We aimed to recruit three age groups (6–9 years, 10–14 years and 15–18 years), resulting in a total sample size of 315.

All statistical analyses were performed using SAS^®^ procedures (version 9.4, Cary, NC, USA). The significance level was set to *p* < 0.05. All data were checked for plausibility and outliers.

Body mass index (BMI [kg/m^2^]) was calculated as the body weight (kg) divided by the square of the body height (m). Sex- and age-independent BMI standard deviation scores (BMI-SDS) were computed using the German national reference data [36].

Socioeconomic status (SES) was assessed using the Winkler index, according to a German representative study among children [37]. This index combines three social status scores: education, profession and total net household income (1–7 points, each). The higher score of either the mother or the father was used as the family socioeconomic status index and categorized into low (3–8 points), middle (9–14 points) or high (15–21 points) social status.

Urbanicity was classified into rural (<5000 inhabitants), small-size urban (5000–<20,000 inhabitants), medium-size urban (20,000–<100,000 inhabitants) or metropolitan (≥100,000 inhabitants).

Dietary intakes were expressed as % of energy intake (%E) or density (intake per 1000 kcal). Intakes of energy, vitamins and minerals were additionally calculated as % of German reference values [38].

Outliers of biomarkers were winsorised (folate *n* = 1, holoTC *n* = 29, LDL-C *n* = 1). The prevalence of biomarker concentrations below or above the respective reference values was calculated.

Sample characteristics in the tables are presented as means ± SD or median (Q1; Q3) for continuous variables. Categorical variables are presented as frequencies and percentages. Differences in categorical characteristics between diet groups were tested using a Chi^2^ test or Fisher’s exact test. For continuous characteristics, Kruskal–Wallis tests for non-parametric data were applied. An analysis of covariance (PROC GLM in SAS) was run to evaluate the group difference between vegetarian, vegan and omnivore participants.

All models were adjusted for sex (boys/girls), age of participants (years), BMI-SDS, SES (high/middle/low), physical activity (MET-minutes), NEM (yes/no) and smoking in the household (yes/no). The 25-OH vitamin D3 model was additionally adjusted for the season of blood withdrawal (April to September/October to March).

Furthermore, the association of duration of exposure to a vegetarian or vegan diet with holoTC and MMA concentrations was analysed among vegetarian and vegan participants using an analysis of covariance.

Missing data among covariates were replaced by the median value of the respective subgroup (puberty *n* = 4, physical activity *n* = 1, smoking in the household *n* = 17, SES *n* = 18, NEM intake *n* = 18). 

To fulfil the model assumption (normal distribution of residuals, homoscedasticity), dietary intake data and biomarker data were transformed (logarithm or square root). An η^2^ ≥ 0.01 is interpreted as small, η^2^ ≥ 0.06 as medium and η^2^ ≥ 0.14 as large effect size [39].

*p*-values were adjusted for multiple testing using the False Discovery Rate method (Proc Multitest in SAS).

Sensitivity analyses of dietary intake models were conducted excluding records categorized as underreported. Sensitivity analyses of biomarker models were conducted, excluding participants consuming food or beverages in the morning before blood withdrawal.

## 3. Results

### 3.1. Sample Characteristics

Of the *n* = 829 children and adolescents registered via recruitment questionnaire, *n* = 522 (63%) met all inclusion criteria and gave the informed consent to participate in the study. Of these, *n* = 121 (23%) participants failed to attend the study centre, e.g., due to schedule difficulties (Supplementary Figure S1). Hence, the final sample consists of *n* = 401 participants (42.9% boys) of which 390 subjects (97%) provided a dietary record, 396 (99%) provided a blood sample and 384 subjects (96%) completed the online questionnaire.

The mean age of participants was 12.7 ± 3.9 years and age ranged from 5.5 years to 19.1 years. The mean age did not differ between the diet groups, as did height, body weight and BMI-SDS (Table 1). The mean BMI-SDS was below zero in all diet groups. On average, vegetarian subjects stated that they had been following this diet for five years, the vegan subjects for more than four years (Table 1).

Slightly more than half of the children and adolescents (*n* = 201; 52.4%) reported the use of dietary supplements. The proportion was highest among vegan participants, followed by vegetarian participants. Of the omnivore participants, less than 17% used dietary supplements (*p* = 0.0012) (Table 1). Vitamin B12 was the nutrient supplement reported most often (vegetarian: 39, vegan: 88%, omnivore: 10%), followed by vitamin D (vegetarian: 27%, vegan: 54%, omnivore: 11%). Iron was supplemented by 20% of vegetarian, 15% of vegan and 2% of omnivore participants. For folate, the prevalence of supplementation was 8% (vegetarian), 11% (vegan) and 4% (omnivore). Between groups, the frequency of supplementation differed significantly for each of these nutrients (*p* < 0.0001) (data not shown).

The rate of underreporting did not differ between diet groups (Table 1), but significantly more records from older subjects were classified as underreported (6 to 9 years: 9.3%, 10 to 14 years: 15.6%, 15 to 18 years: 29.7%, *p* < 0.0001, data not shown).

About three quarters of the subjects (*n* = 280; 73.1%) came from families with a high SES and about half of the sample lived in a large city (*n* = 189; 50.5%). Smoking in the household was uncommon in the total sample (<10%).

Among the completed questionnaires on puberty (four missing data), 39.3% (*n* = 156) indicated Tanner stage 1 (pre-puberty), 42.1% (*n* = 167) indicated at least one criterion of Tanner stage 2–4 (puberty) and 18.6% (*n* = 74) at least one criterion for Tanner stage 5 (post-puberty). Participants reported around three hours of physical activity per week. Differences between the diet groups were not significant (Table 1).

### 3.2. Energy and Nutrient Intake

#### 3.2.1. Energy Intake

The mean total energy intake was around 7 MJ/day (1630–1700 kcal/day) (Table 2) and was in the range of energy requirements for low physical activity (Supplementary Figure S2). The total energy intake did not differ between groups (Table 2), but the median energy density (kJ/g) was significantly lower among the vegan group compared to the vegetarian group (*p* = 0.0039) and tended to be lower than among the omnivore group (*p* = 0.0512).

#### 3.2.2. Macronutrient Intake

The median protein intake exceeded the German reference values of 0.9 g/kg body weight/day in all diet groups and was lowest among vegetarian participants. The intake of carbohydrates was higher in vegetarians and vegans than among omnivore subjects on average (*p* = 0.0002, respectively). Vegan participants reported a lower mean intake of free sugar than vegetarian and omnivore (*p* = 0.0002, respectively). Effect sizes of differences among carbohydrates and free sugars were medium. Dietary fibre intake was highest among vegans and lowest among omnivore participants, and the effect size was large. The median fat intake (%E) was below 30%E among vegan participants; the fat intake of vegetarians and omnivores was significantly higher (*p* < 0.04). The intake of SFA (%E) was highest among omnivores and lowest among vegans (*p* = 0.0002), with intermediate intakes among vegetarian participants on average. In contrast, vegan participants had the highest intake of PUFA (*p* = 0.0002). The effect sizes of group differences of SFA and PUFA intake were large.

#### 3.2.3. Micronutrient Intake

The mean intakes of all vitamins except vitamin A (retinol-equivalents) and minerals differed significantly between the groups (expressed per 1000 kcal) (Table 2). Vegan participants had the highest intake of vitamin E (tocopherol-equivalents), magnesium and iron, whereas omnivore participants had the lowest intake of these nutrients, with intermediate values among vegetarian participants (*p* < 0.01, Table 2). Instead, the intake of calcium was highest in omnivores and vegetarians, but lowest in vegans (*p* < 0.02). In addition, the median intakes of vitamin C and folate-equivalents did not differ between vegetarian and omnivore participants. Intake of these vitamins was highest in the vegan group (*p* < 0.04 and *p* = 0.0002). Highest median intakes of vitamin B2 were found among omnivores, and lowest among vegans with intermediate values among vegetarians (*p* < 0.02). Large effect sizes of diet group differences were found for vitamin B12, magnesium and iron. The sensitivity analysis excluding 128 dietary records classified as underreported largely confirmed these results (Supplementary Table S1).

The median intake of vitamin C and magnesium exceeded reference values independent of the diet group (Supplementary Figure S2a,b). Moreover, the median intakes of vitamin A, vitamin E, vitamin B1 folate, iron and zinc reached at least 80% of the reference values, respectively. The median calcium intake of vegan participants was <50% of the reference value (vegetarian: 56%, omnivore: 67%). The median intake of vitamin B2 was around 60% of the reference value (vegetarian: 75%, omnivore: 83%). The vitamin B12 intake of vegan participants only reached 4% of reference values (vegetarian: 32%, omnivore: 86%). Significant differences between vegetarians and omnivores were only found for vitamin E and vitamin B12. Vegan participants had the highest intake compared to the reference values of vitamin E, vitamin B1, folate, vitamin C, magnesium, iron and zinc, but the lowest intake of vitamin B2 and vitamin B12 (Supplementary Figure S2a,b).

### 3.3. Nutrient Status and Blood Lipids

There was no significant difference of haemoglobin, vitamin B2 (FAD), 25-OH vitamin D3, HDL-C and TG blood concentrations between diet groups (Table 3). Ferritin concentration was significantly higher in omnivore participants than in vegetarian (*p* = 0.0134) and vegan (*p* = 0.0404) participants.

Vegan participants had higher folate concentrations than vegetarian participants (*p* = 0.0053). Vegetarian participants had lower holoTC (*p* = 0.0042) and higher MMA concentrations (*p* = 0.0253) than omnivore participants.

TC was lower among vegan than among vegetarian (*p* = 0.0065) and omnivore (*p* = 0.0010) participants. Vegan participants had the lowest non-HDL-C and LDL-C concentrations in comparison to vegetarian (*p* = 0.0053 and *p* = 0.0041) and omnivore (*p* = 0.0010 and *p* = 0.0010) participants. However, the effect sizes of all differences were small.

A sensitivity analysis excluding those subjects who had reported that they had consumed caloric foods or beverages before the blood withdrawal (*n* = 31) yielded largely identical results (Supplementary Table S2).

There was no significant association of duration of exposure to a vegetarian or vegan diet with MMA or holoTC concentrations, either in the total sample, nor stratified by diet group (*p* > 0.05, data not shown).

Overall prevalence of haemoglobin, ferritin and folate concentrations below reference values was low (Supplementary Figure S3a). Prevalence of holoTC < 50 pmol/L ranged between 24% (omnivore participants) and 39% (vegetarian participants). High MMA concentrations were found in 14% of vegetarian participants, 11% of vegan participants and 7% of omnivore participants (Supplementary Figure S3b). Considering both holoTC and MMA concentrations, 13% of vegetarian, 8% of vegan and 4% of omnivore participants were categorized as likely deficient and 80% (vegetarian), 85% (vegan) and 94% as unlikely deficient, respectively (data not shown).

One third of participants showed 25-OH vitamin D3 concentrations below 20 ng/mL (vegetarian: 36%, vegan: 27%, omnivore: 28%), and 7% below 12 ng/mL (vegetarian: 10%, vegan: 5%, omnivore: 4%) (Supplementary Figure S3c). In addition, nearly half of the participants had low concentrations of vitamin B2 (FAD) (vegetarian: 50%, vegan: 54%, omnivore: 37%). Moreover, the majority of participants had acceptable concentrations of TC (vegetarian: 81%, vegan: 89%, omnivore: 70%), HDL-C (vegetarian: 77%, vegan: 79%, omnivore: 84%), LDL-C (vegetarian: 89%, vegan: 93%, omnivore: 79%), non-HDL-C (vegetarian: 93%, vegan: 95%, omnivore: 85%) and TG (vegetarian: 72%, vegan: 72%, omnivore: 85%) (Supplementary Figure S3d).

## 4. Discussion

The cross-sectional VeChi Youth Study did not indicate specific nutritional risks among vegetarian and vegan compared to omnivore children and adolescents. Iron and folate status was sufficient in the majority of the total sample. Biomarker concentrations of 25-OH vitamin D3 and vitamin B2 were low in a notable proportion of the study sample independent from the diet group. Although reported intake of vitamin B12 from food was far below the dietary reference among vegan participants, a deficiency of this vitamin was unlikely among the majority of subjects, according to biomarker concentrations. The median calcium intake was also below the reference, irrespective of the diet group, although it was lowest for the vegan group. Blood lipids were mostly within an acceptable range, with the lowest concentrations of TC, non-HDL-C and LDL-C among the vegan group.

Vitamin B12 is regarded as the most critical nutrient in vegan diets, since only foods of animal origin are natural sources of this vitamin [40]. In Germany, vitamin B12 fortification of vegan food products, e.g., juices or dairy alternatives, is uncommon [41]. Hence, the observed low intake of this vitamin among vegan participants had to be expected. Nevertheless, the results of the biomarker analysis (holoTC, MMA) did not indicate a vitamin B12 deficiency in the majority of vegan participants. Obviously, the use of vitamin B12 supplements was sufficient to ensure an adequate vitamin B12 status for the majority (>90%) of the vegan children and adolescents in our sample, even with a mean duration of a vegan diet of four years. It should be noted that the prevalence of supplementation of vitamin B12 was considerably higher than among vegetarian and vegan participants of a recently published study with Polish 5–10 year old children (vegetarian: 35%, vegan 44%) [14]. However, in our sample there was a tendency toward an insufficient vitamin B12 supply among the vegetarian subgroup. In addition, a review on vitamin B12 status measured by serum vitamin B12 showed considerable proportions of deficiency not only among vegan, but also among vegetarian subjects [42]. Whether this trend in vegetarians is mainly due to the lack of vitamin B12 intake via meat and fish, or also caused by a reduced intake of dairy products, remains to be investigated. Therefore, vitamin B12 supplementation should not only be encouraged among vegan, but also among vegetarian children and adolescents.

Vitamin B2 (FAD) and 25-OH vitamin D3 were the nutrients with the most prevalent blood concentrations below cut-off values. To the best of our knowledge, there are no studies on the vitamin B2 status of vegetarian or vegan children or adolescents. Among adults, there was no significant difference of blood vitamin B2 concentrations and percentage of subjects below the cut-off in a sample of 53 vegan, 53 vegetarian and 100 omnivore subjects from Switzerland [43]. In Austria, about 30% of vegan participants were considered to be vitamin B2 deficient, compared to only 10% of vegetarian and omnivore participants [44]. In Germany, vitamin B2 concentrations were lower among vegan than omnivore adults, but median concentration was above the reference value [45]. Although the biochemical thresholds for deficiency may be inappropriate [46] and the clinical relevance of the low vitamin B2 (FAD) concentrations observed in the VeChi Youth Study is still unclear, vegan, vegetarian and also omnivore children and adolescents should optimize their vitamin B2 intake. Besides dairy products as a main source for vitamin B2, there are several plant sources for this vitamin, e.g., nuts, mushrooms, legumes, textured vegetable protein (TVP) or fortified plant-based dairy alternatives.

Concentrations of 25-OH vitamin D3 did not differ significantly between the diet groups, although the prevalence of supplementation was highest in the vegan group. However, the prevalence of low concentrations was high, as in representative studies in Germany [47], partly because in Germany, unlike for example in the USA [6], supplementation is not recommended for children even with low vitamin D intakes. The aforementioned Polish study confirmed the relevance of vitamin D supplementation in children consuming plant-based diets [14]. However, vitamin D is strongly associated with calcium absorption and metabolism. In the VeChi Youth Study, median calcium intake was below the reference values in the whole sample, which is in accordance with a recent German representative study [48].

Mean calcium intake was lowest in the vegan group with intermediate values in the vegetarian subgroup. Studies indicate that vegetarian, including vegan, adults have a lower bone mineral density [49] and a higher risk for fractures [50]. Two cross-sectional studies confirmed a lower bone mineral density among vegetarian and/or vegan children [12,14]. However, in addition to calcium and vitamin D, several other dietary and non-dietary factors affect bone health and the relationship of vegetarian and vegan food patterns and bone health is complex and not yet fully understood. In any case, vegetarian and vegan children and adolescents are encouraged to achieve adequate calcium intakes to avoid detrimental effects on bone health [51]. Besides dairy products as the major calcium source in Western vegetarian and omnivore diets, plant food sources are dark green vegetables with low oxalate contents (e.g., kale, bok choy, broccoli), tofu, almonds and other tree nuts, dried figs, chickpeas, calcium-rich mineral water (≥400 mg/L) and calcium-fortified dairy alternatives (e.g., soy or oat milk). Furthermore, an adequate vitamin D status is important to ensure a sufficient intestinal calcium absorption, in particular when consuming a vegan diet with low calcium intakes. Hence, vitamin D supplementation should be encouraged among vegan individuals to optimize absorption of dietary calcium.

In the VeChi Youth Study, omnivore children had the highest median protein intake; however, the median protein intake of vegetarian and vegan participants was also adequate. Still, due to the lower protein quality of plant protein, a higher protein requirement (+15–20%) among vegan children and adolescents has been proposed [52]. On the other hand, energy requirements during growth are very high compared to those of protein. Furthermore, a blend of plant food proteins is consumed in a vegan diet. Hence, the concern of inadequate protein or amino acid intake seems not to be relevant in practice [53]. In a recent untargeted metabolomics analysis among a small group of Finnish toddlers, the vegan group had a pattern of overall lower circulating concentrations of essential amino acids, but only few individual amino acids concentrations differed significantly [17]. Such results have to be regarded against the background of overall high protein intakes in the general paediatric population [54,55].

In contrast to a recent study [14], there was no elevated risk for iron deficiency in the vegetarian or vegan subgroup. Obviously, the higher iron intake observed in the vegan participants of the VeChi Youth Study could compensate for the lower bioavailability of non-haem iron in plant-based diets (average iron bioavailability is 5–12% in a vegetarian diet, 14–18% in a mixed diet) [56,57]. Furthermore, iron was supplemented more often in vegetarian or vegan participants than in omnivore participants. This is in accordance with a review on iron status among vegetarian children [58] and a recent study comparing the nutrient status of vegan and omnivore adults in Germany [45]. Median ferritin concentrations were lower in vegan than in vegetarian and omnivore participants of the VeChi Youth Study. However, the prevalence of concentrations below the cut-off was small. Hence, the observed diet group differences appear to be of minor importance.

Since the focus of many statements on vegan diets is on potential health risks, it should be emphasized that vegan participants in our study had the highest intakes of vitamin E, vitamin B1, folate, vitamin C, magnesium, iron and zinc. Intake of free sugars was lowest in this group, although total carbohydrate intake was high. The median intake of vegetarian and omnivore participants slightly exceeded the limit of 10%E from free sugars suggested by the WHO [59]. However, compared to other studies [60], the level of free sugar intake was low in all diet groups indicating a health-conscious sample. The mean fibre intake was highest among vegan compared to vegetarian and omnivore participants. The latter did not reach the dietary reference value of 14 g/1000 kcal [61].

The median fat intake was lowest among vegan participants, and this subgroup also showed the best dietary fat quality indicated by the lowest intake of SFA and the highest intake of PUFA. Accordingly, vegan participants had the lowest serum TC, LDL-C and non-HDL-C concentrations. This result is in accordance with the abovementioned recent German study on vegan and omnivore adults [45]. Studies with adult vegetarian and vegan participants showed, accordingly, a lower risk for most prevalent non-communicable diseases, e.g., dyslipidaemia, atherosclerosis and coronary heart disease [62,63]. The VeChi Youth Study provides the first evidence of reduced cardiovascular risk factors already in childhood and adolescence, only with a vegan diet. The lack of significant differences between vegetarian and omnivore participants could be explained by the young age and low-risk profile of both groups.

There are some strength and limitations of the VeChi Youth Study. First, a major strength is the large sample size and the use of biomarkers to assess nutritional status. Other strengths are the measurement of anthropometrics by trained staff, the relative balance of the study groups with no significant differences in age and sociodemographic characteristics and the differentiation between vegetarian and vegan subjects. The VeChi Youth Study’s diet group definition takes into account that people who describe themselves as vegetarian or vegan do not always consistently avoid meat and fish or all animal foods [64]. A further strength is the detailed dietary assessment. The higher proportion of underreporting among older participants is well known in the literature. Potential reasons are that parents assisting younger children during recording may provide more valid responses than the unsupervised recording of an adolescent. Furthermore, adolescents are prone to more unstructured eating patterns and eating out-of-home [65]. The major limitations are the cross-sectional design of the study and the lack of representativity. However, the high SES of the VeChi Youth Study sample (three-quarters with a high Winkler index) and the place of residence in a (large) city correspond to the known socio-demographic characteristics of vegetarian and vegan individuals in Germany. The significant proportion of omnivore participants from families with a high SES is probably due to a higher willingness to participate in nutritional studies in this population subgroup, which was also observed in other studies [14,66,67]. However, this lack of representativity has to be kept in mind when interpreting the results, and it restricts the generalizability of the results.

## 5. Conclusions

The results of the VeChi Youth Study confirms the position of several national nutrition or paediatric societies [68,69,70,71] that a vegetarian, including a vegan, diet can meet the recommended nutrient requirements in childhood and adolescence. However, due to the cross-sectional design, the VeChi Youth Study only provides a glimpse of plant-based diets and health in these age groups. Hence, follow-ups of our study sample are desirable to examine the long-term health impacts of vegetarian and vegan diets in children and adolescents, in particular with respect to bone health. Furthermore, other potential critical nutrients should be examined, e.g., intake and status of indispensable amino acids, long-chain n3-fatty acids, iodine and selenium. The results are not readily transferable to other, especially younger, age groups. Particularly for infants, special recommendations apply [72].

## Figures and Tables

**Table 1:** Sample characteristics of the VeChi Youth Study (*n* = 401, 6–18 years of age).

	Vegetarian (*n* = 150)	Vegan (*n* = 114)	Omnivore (*n* = 137)	*p* ^1^
Boys	59 (34.3)	38 (22.1)	75 (43.6)	
Girls	91 (39.7)	76 (33.2)	62 (27.1)	
Age (years)	12.6 ± 3.9	12.9 ± 4.2	12.6 ± 3.7	0.7766
Height (cm)	154 ± 20	152 ± 19	156 ± 20	0.4902
Weight (kg)	45 ± 18	43 ± 16	46 ± 17	0.4902
BMI-SDS	−0.3 ± 0.9	−0.6 ± 0.9	−0.3 ± 1.0	0.1506
*Dietary variables*				
Exposure to diet (years) ^2^	5.0 ± 3.9	4.2 ± 3.4	n.a.	0.1506
Use of dietary supplements ^3^	74 (52.1)	105 (95.5)	22 (16.8)	0.0012
Underreporting ^4^	31 (21.4)	18 (16.4)	23 (17.0)	0.5159
Consuming caloric food or drink before blood withdrawal ^5^	15 (10.0)	9 (8.1)	7 (5.2)	0.4297
*Sociodemographic variables*				
Socioeconomic status ^6^
High	106 (74.1)	68 (62.4)	106 (80.9)	
Middle	34 (23.8)	37 (33.9)	25 (19.1)	
Low	3 (2.1)	4 (3.7)	0 (0)	0.0456
Urbanicity ^7^				
Large city	71 (51.8)	59 (54.1)	59 (46.1)	
Medium sized	48 (35.0)	32 (29.4)	48 (37.5)	
Small city	6 (4.4)	11 (10.1)	13 (10.2)	
Rural community	12 (8.8)	7 (6.4)	8 (6.3)	0.5216
Smoking in the household (never) ^8^	136 (95.1)	103 (93.6)	128 (97.7)	0.4902
*Puberty* ^9^				
Pre-pubertal	57 (39.0)	44 (38.6)	55 (40.2)	
Pubertal	64 (43.8)	43 (37.7)	60 (43.8)	
Post-pubertal	25 (17.1)	27 (23.7)	22 (16.1)	0.6110
*Physical Activity* ^10^				
Activity hours	3.1 (1.7)	3.2 (2.0)	3.3 (1.8)	0.4902
MET-minutes	1212 (724)	1188 (815)	1315 (775)	0.5830

^1^ Chi^2^-test or Fisher’s exact test (categorical variables) or Kruskal–Wallis test or *T*-test (continuous variables), *p*-values adjusted for multiple testing according to the False Discovery Rate method. ^2^ Exposure to diet: missing *n* = 16 (vegetarian *n* = 10, vegan: *n* = 6). ^3^ Use of supplements: missing *n* = 18 (vegetarian: *n* = 8; vegan: *n* = 4; omnivore: *n* = 6). ^4^ Underreporting: missing *n* = 11 (vegetarian: *n* = 5, vegan: *n* = 4, omnivore: *n* = 2). ^5^ Blood sampling was unsuccessful among five participants (vegetarian: *n* = 1, vegan: *n* = 3, omnivore: *n* = 1. ^6^ High social class: Winkler index > 14, middle social class: Winkler index > 9 to 14, low social class: Winkler index ≤ 9, 18 missing (vegetarian: *n* = 8, vegan: *n* = 4; omnivore: *n* = 6). ^7^ Large city: >100,000 inhabitants, medium sized city: 20,000 to <100,000 inhabitants, small city: 5000 to <20,000 inhabitants, rural community: <5000 inhabitants; 17 missing, 10 “don’t know”, (vegetarian: *n* = 14; vegan: *n* = 4; omnivore: *n* = 9). ^8^ Smoking in the household missing *n* = 17 (vegetarian: *n* = 7; vegan: *n* = 4, omnivore: *n* = 6). ^9^ Puberty missing *n* = 4 (only vegetarian). ^10^ Physical activity missing *n* = 1 (only vegan).

**Table 2:** Energy and nutrient intake (including fortification, without supplements) of vegetarian (VG, *n* = 145), vegan (VN, *n* = 110) and omnivore (OM, *n* = 135) participants of the German VeChi Youth Study (*n* = 390, 6–18 years old) ^1^.

	VG	VN	OM	Total Model	Pairwise Comparison		
	P50 (P25; P75)	P50 (P25; P75)	P50 (P25; P75)	*p* ^2^ [*η*^2^]	VG-VN *p* ^2^	VG-OM *p* ^2^	VN-OM *p* ^2^
Energy (kcal/day)	1708 (1367; 1975)	1634 (1358; 1903)	1737 (1431; 2150)				
(MJ/day)	7.2 (5.7; 8.3)	6.8 (5.7; 8.0)	7.3 (6.0; 9.0	0.9922 [0.0001]	0.9366	0.9922	0.9922
ED (kJ/g) ^3^	5.94 (5.11; 7.08)	5.39 (4.68; 6.14)	6.16 (5.26; 7.19)	0.0152 [0.0238]	0.0039	0.6110	0.0512
*Macronutrients*							
Protein (g/kg BW/day)	1.14 (0.88; 1.53)	1.16 (0.89; 1.67)	1.36 (1.07; 1.74)	0.0011 [0.0386]	0.0180	0.0011	0.5918
Carbohydrates (%E)	54.7 (50.2; 59.3)	56.5 (50.6; 61.2)	49.1 (45.0; 54.6)	0.0002 [0.0679]	0.2994	0.0002	0.0002
Free sugars (%E) ^4^	11.6 (8.1; 15.4)	6.6 (4.0; 9.5)	10.5 (7.3; 15.5)	0.0002 [0.0929]	0.0002	0.1789	0.0002
Dietary fibre (g/1000 kcal)	14.7 (12.0; 17.7)	21.9 (18.0; 25.5)	12.0 (10.1; 14.2)	0.0002 [0.2082]	0.0002	0.0006	0.0002
Fat (%E)	32.3 (28.0; 37.8)	29.4 (25.3; 36.6)	36.4 (30.7; 40.6)	0.0037 [0.0316]	0.0368	0.0376	0.0010
SFA (%E)	12.5 (9.9; 15.6)	7.8 (5.9; 10.3)	15.9 (12.9; 18.8)	0.0002 [0.1888]	0.0002	0.0002	0.0002
MUFA (%E)	10.3 (8.7; 12.3)	9.5 (7.6; 13.0)	11.8 (10.2; 14.0)	0.0008 [0.0282]	0.1370	0.0178	0.0022
PUFA (%E)	6.1 (4.7; 7.9)	8.6 (7.0; 10.8)	4.8 (3.9; 6.0)	0.0002 [0.1556]	0.0002	0.0002	0.0002
*Vitamins*							
Retinol-Equivalents (µg/1000 kcal)	435 (317; 641)	465 (330; 698)	453 (337; 650)	0.2719 [0.0076]	0.9175	0.1116	0.2759
Tocopherol-Equivalents (mg/1000 kcal)	7.2 (5.7; 9.4)	9.6 (7.9; 11.6)	6.0 (4.8; 7.6)	0.0002 [0.0978]	0.0002	0.0015	0.0002
Vitamin C (mg/1000 kcal)	45 (31; 64)	67 (43; 91)	44 (30; 66)	0.0015 [0.0361]	0.0004	0.2731	0.0398
Folate-Equivalents (µg/1000 kcal)	191 (101; 147)	152 (126; 185)	109 (83; 131)	0.0002 [0.0623]	0.0002	0.0768	0.0002
Vitamin B1 (µg/1000 kcal)	440 (360; 558)	605 (497; 700)	465 (413; 560)	0.0002 [0.0927]	0.0002	0.0413	0.0012
Vitamin B2 (µg/1000 kcal)	476 (382; 588)	381 (304; 483)	544 (458; 645)	0.0002 [0.0754]	0.0002	0.0149	0.0002
Vitamin B12 (µg/1000 kcal)	0.6 (0.4; 1.1)	0.1 (0.0; 0.2)	1.6 (1.2; 2.0)	0.0002 [0.2057]		0.0002	
*Minerals*							
Calcium (mg/1000 kcal)	390 (300; 494)	305 (236; 424)	400 (330; 474)	0.0011 [0.0266]	0.0026	0.9247	0.0182
Magnesium (mg/1000 kcal)	176 (153; 210)	251 (206; 305)	153 (135; 179)	0.0002 [0.2220]	0.0002	0.0216	0.0002
Iron (mg/1000 kcal)	6.8 (5.6; 7.8)	9.2 (7.6; 10.8)	5.7 (5.2; 6.6)	0.0002 [0.1922]	0.0002	0.0099	0.0002
Zinc (mg/1000 kcal)	4.7 (3.9; 5.3)	5.1 (4.3; 6.0)	5.0 (4.4; 5.6)	0.0002 [0.531]	0.0002	0.0137	0.1680

Unadjusted data, BW: Body weight; %E: Percentage of energy intake. ^1^ Eleven participants did not provide a dietary record. ^2^ Analysis of covariance, adjusted for age (years), BMI-SDS, socioeconomic status (low/middle/high), smoking in the household (yes/no), physical activity (MET-minutes), use of dietary supplements (yes/no), *p*-values were adjusted for multiple testing according to the False Discovery Rate (FDR) method. ^3^ Energy density, excluding beverages. ^4^ Added sugars as well as sugars from juices.

**Table 3:** Nutrient biomarker and blood lipids of vegetarian (vegetarian, *n* = 149), vegan (vegan, *n* = 111) and omnivore (OM, *n* = 136) participants of the German VeChi Youth Study (*n* = 396, 6–18 years old) ^1^.

	VG	VN	OM	Total Model	Pairwise Comparison		
	P50 (P25; P75)	P50 (P25; P75)	P50 (P25; P75)	*p* ^2^ [*η*^2^]	VG-VN *p* ^2^	VG-OM *p* ^2^	VN-OM *p* ^2^
*Nutrient biomarkers*							
Haemoglobin (g/dL)	13.3 (12.4; 14.1)	13.2 (12.5; 14.0)	13.5 (12.8; 14.2)	0.6520 [0.0029]	0.9879	0.4143	0.5144
Ferritin (µg/L)	29 (20; 39)	29 (22; 42)	38 (26; 52)	0.0312 [0.0235]	0.8081	0.0134	0.0404
25-OH Vitamin D3 (ng/mL)	23 (17; 31)	26 (20; 33)	24 (18; 31)	0.3519 [0.0074]	0.7702	0.1704	0.4143
Vitamin B2 (FAD) (µg/L)	199 (175; 223)	197 (169; 215)	206 (190; 225)	0.2648 [0.0094]	0.4518	0.2423	0.1284
Folate (µg/L)	279 (251; 320)	319 (287; 363)	284 (252; 327)	0.0134 [0.0286]	0.0053	0.3366	0.1561
HoloTC (pmol/L)	56 (41; 83)	70 (44; 111)	67 (50; 86)	0.0120 [0.0304]	0.3519	0.0042	0.1788
MMA (nmol/L)	153 (127; 203)	144 (110; 178)	153 (119; 195)	0.0701 [0.0183]	0.6716	0.0253	0.1733
*Blood lipids*							
TC (mg/dL)	144 (131; 162)	133 (120; 150)	153 (134; 173)	0.0016 [0.0429]	0.0065	0.1200	0.0010
HDL-C (mg/dL)	54 (46; 68)	53 (47; 64)	57 (48; 66)	0.6716 [0.0024]	0.6538	0.5950	0.4288
Non-HDL-C (mg/dL)	89 (74; 100)	78 (63; 94)	96 (73; 113)	0.0010 [0.0458]	0.0053	0.1003	0.0010
LDL-C (mg/dL)	79 (69; 93)	68 (57; 84)	90 (70; 106)	0.0010 [0.0514]	0.0041	0.0701	0.0010
TG (mg/dL)	70 (55; 91)	69 (53; 85)	61 (51; 80)	0.1283 [0.0144]	0.4185	0.0573	0.4288

Unadjusted data, holoTC: holotranscobalamin, MMA: methylmalonic acid, TC: total cholesterol, TG: triglycerides. ^1^ Five participants did not provide a blood sample, and further missing values resulted from an insufficient sample volume or technical problems during the analysis (haemoglobin and folate *n* = 3, ferritin *n* = 1, vitamin B2 (FAD) *n* = 4, holoTC *n* = 9, MMA *n* = 2). ^2^ Analysis of covariance, adjusted for age (years), BMI-SDS, socioeconomic status (low/middle/high), smoking in the household (yes/no), physical activity (MET-minutes), use of dietary supplements (yes/no) and season (only 25-OH vitamin D3), *p*-values were adjusted for multiple testing according to the False Discovery Rate (FDR) method.

## Data Availability

The data described in the manuscript, code book and analytic code will not be made available because of data protection regulations.

## Supplementary Materials

The following are available online at https://www.mdpi.com/article/10.3390/nu13051707/s1, Figure S1: Flow chart of recruitment of vegetarian (VG), vegan (vegan) and omnivore (OM) children and adolescents (6–18 years old) in the VeChi Youth Study, Figure S2: Intake of energy, minerals (a) and vitamins (b) expressed as % of dietary reference values of vegetarian (VG), vegan (VN) and omnivore (OM) participants of the German VeChi Youth Study (*n* = 390, 6–18 years), Figure S3: Prevalence of high, low or normal/acceptable concentrations of nutrient biomarkers and blood lipids among vegetarian (VG), vegan (VN) and omnivore participants of the German VeChi Youth Study (*n* = 396, 6–18 years old). Table S1: Energy and nutrient intake (including fortification, without supplements) of vegetarian (VG), vegan (VN) and omnivore (OM) participants of the German VeChi Youth Study (*n* = 318, 6–18 years old), excluding dietary records classified as underreported, Table S2: Nutrient biomarker and blood lipids of children and adolescents of the German VeChi Youth Study (*n* = 365, 6–18 years old) stratified by diet group, excluding those participants consuming caloric food or drinks before blood withdrawal.
