# Supplementary material for: Nutrient Intake and Status of German Children and Adolescents Consuming Vegetarian, Vegan or Omnivore Diets: Results of the VeChi Youth Study
Source: Nutrients. 2021 May 18;13(5):1707. doi: 10.3390/nu13051707 (PMC8157583; doi:10.3390/nu13051707)
Supplement: Supplementary file 1 [file nutrients-13-01707-s001.zip › nutrients-1212933-supplementary.pdf]

Supplementary figures

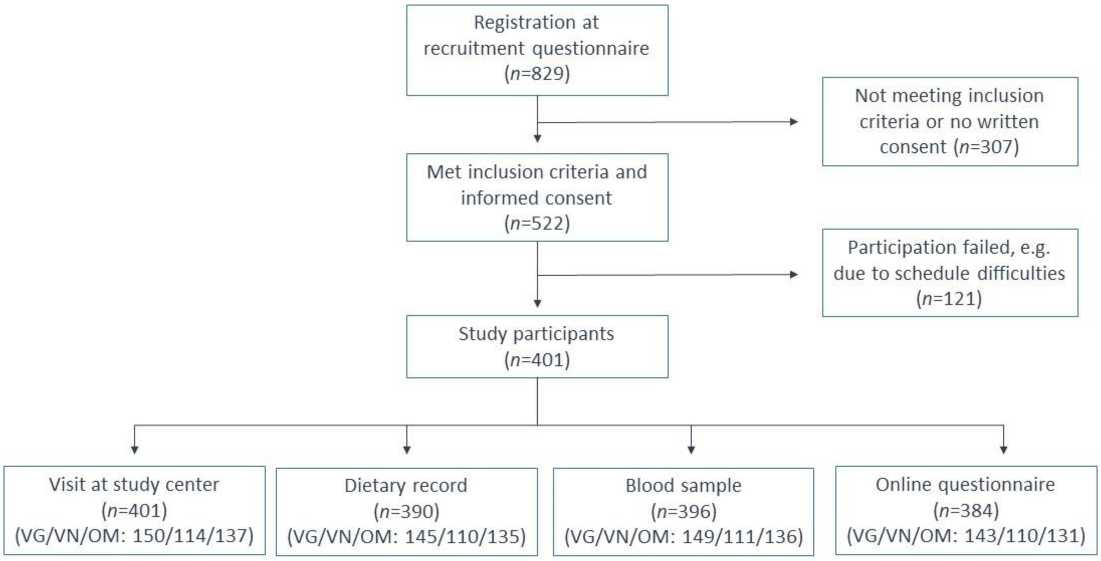

SUPPLEMENTAL FIGURE S1 Flow chart of recruitment of vegetarian (VG), vegan (VN), and omnivore (OM) children and adolescents (6-18 years old) in the VeChi Youth Study

a

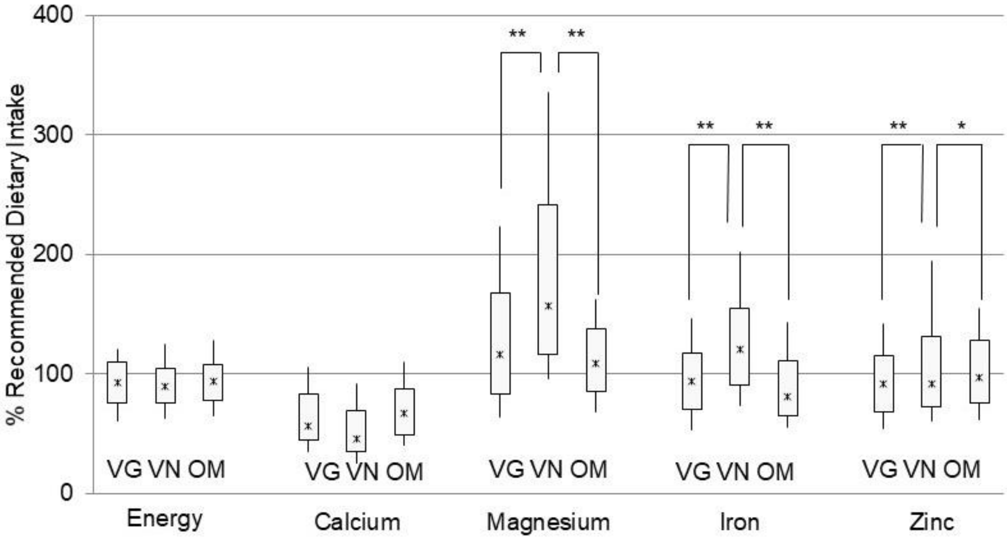

b

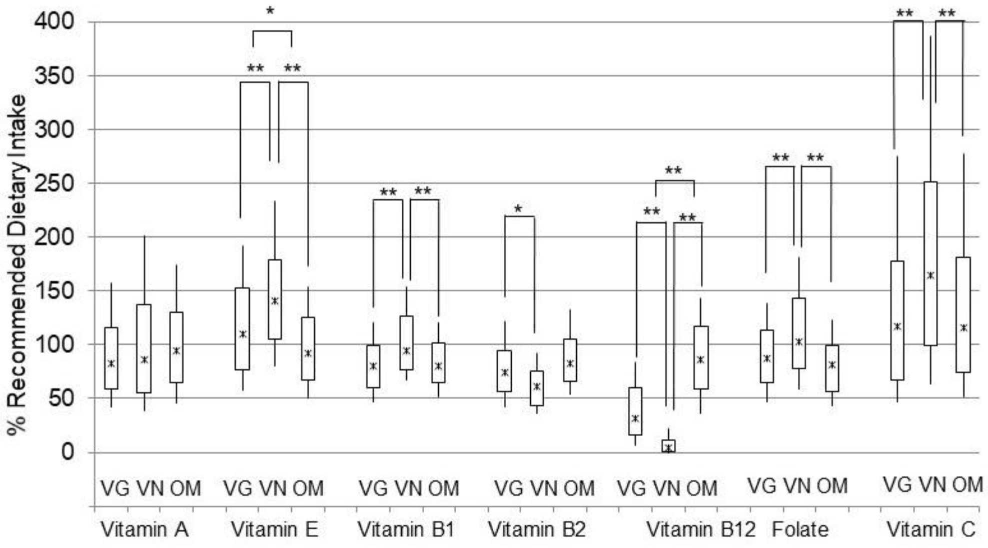

SUPPLEMENTAL FIGURE S2 Intake of energy, minerals (a) and vitamins (b) expressed as % of dietary reference values of vegetarian (VG), vegan (VN), and omnivore (OM) participants of the German VeChi Youth Study (n = 390, 6-18 years); analysis of covariance, adjusted for sex (boys/girls), age of participants (years), BMI-SDS, SES (high/middle/low, physical activity (MET-minutes), supplement intake (yes/no) and smoking in the household (yes/no), adjusted for multiple testing using the False Discovery Rate (FDR) method, ** $P < 0.001$, * $P < 0.01$.

a

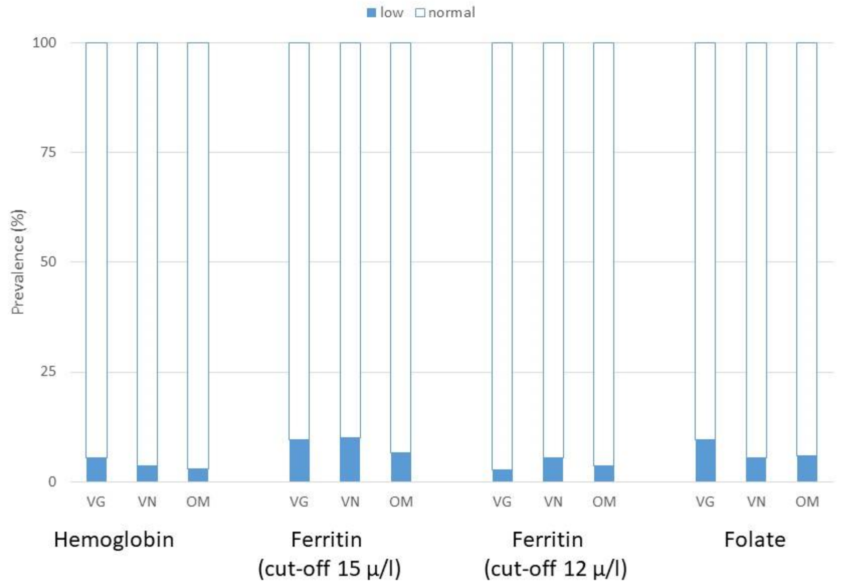
low
normal
100
75
50
25
0
Prevalence (%)
VG
VN
OM
Hemoglobin
Ferritin
(cut-off 15 µ/l)
Ferritin
(cut-off 12 µ/l)
Folate

b

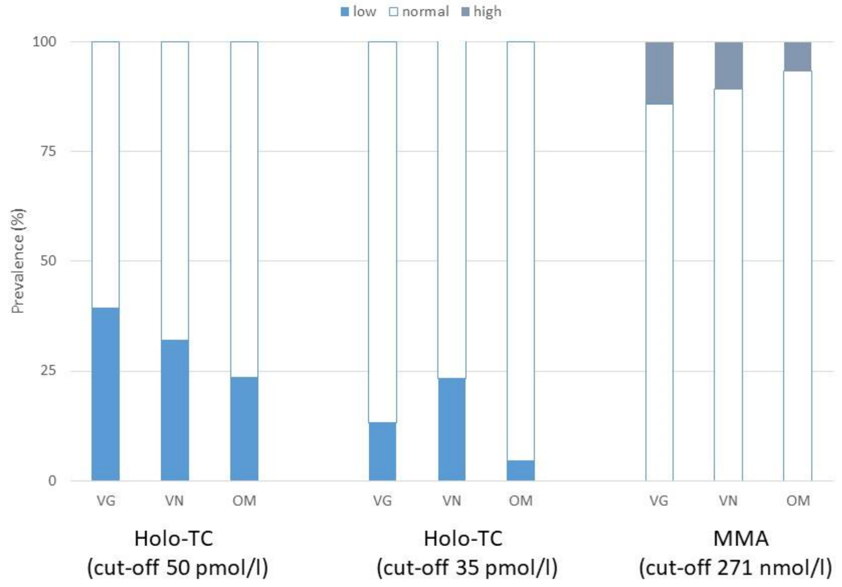
low
normal
high
100
75
50
25
0
Prevalence (%)
VG
VN
OM
Holo-TC
(cut-off 50 pmol/l)
Holo-TC
(cut-off 35 pmol/l)
MMA
(cut-off 271 nmol/l)

c

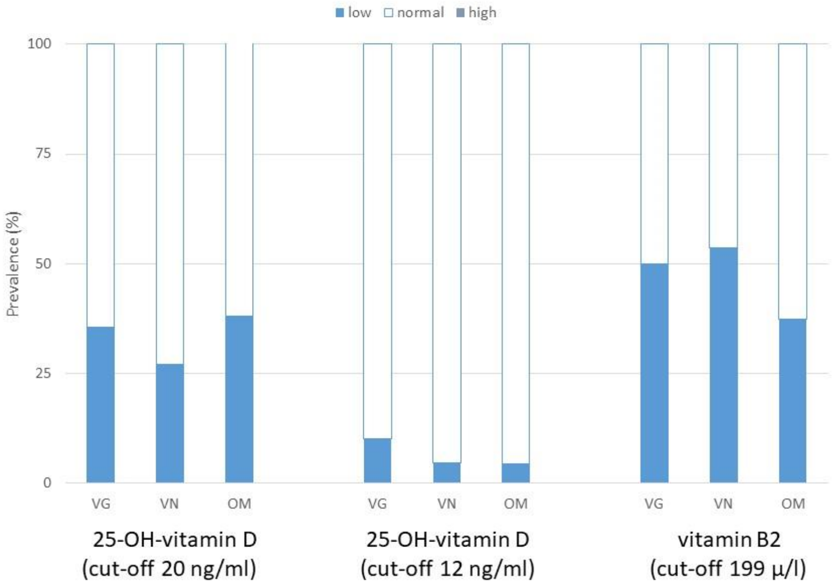

d

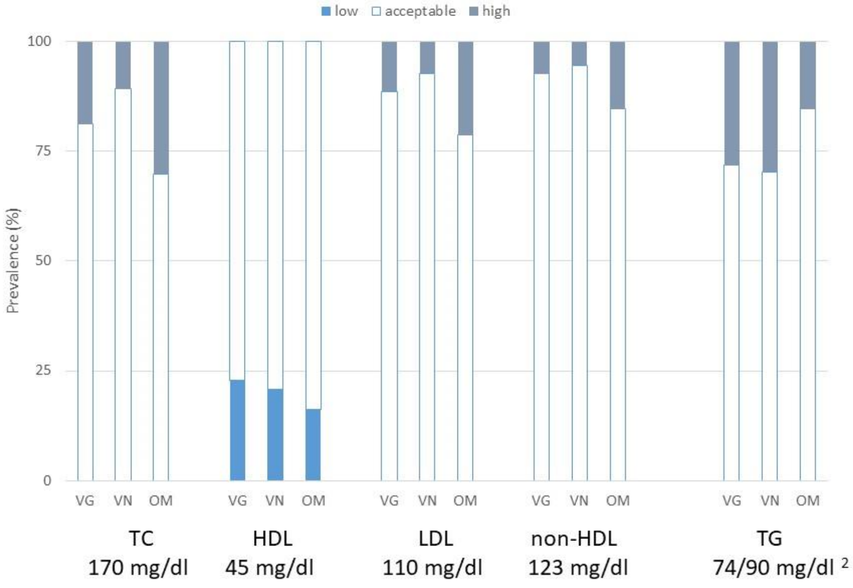

SUPPLEMENTAL FIGURE S3 Prevalence of high, low, or normal/acceptable concentrations of nutrient biomarkers and blood lipids among vegetarian (VG), vegan (VN), and omnivore participants of the German VeChi Youth Study (*n* = 396, 6-18 years old)

[1]6-12 years: <11.5 g/dL, 12-<15 years and girls > 15 years: <12 g/dL, boys > 15 years: <13 g/dL

[2] 6-9 years: 74 mg/dL, 10-19 years: 90 mg/dL

Supplementary Tables

SUPPLEMENTAL TABLE 1 Energy and nutrient intake (including fortification, without supplements) of vegetarian (VG), vegan (VN), and omnivore (OM) participants of the German VeChi Youth Study (*n* = 318[1], 6-18 years old), excluding dietary records classified as underreported

	Vegetarian	Vegan	Omnivore	Total model	Pairwise comparison		
	(*n* = 114) P50 (P25; P75)	(*n* = 92) P50 (P25; P75)	(*n* = 112) P50 (P25; P75)	P^2	VG-VN P^2	VG-OM P^2	VN-OM P^2
Energy (kcal/day)	1829 (1534; 2072)	1675 (1465; 1951)	1901 (1539; 2306)	0.7152	0.4318	0.9007	0.5862
(MJ/day)	7.7 (6.4; 8.7)	7.0 (6.1; 8.2)	8.0 (6.4; 9.7)				
Energy density (kJ/g) [3]	6.1 (5.2; 7.0)	5.5 (4.8; 6.2)	6.4 (5.6; 7.2)	0.1175	0.0667	0.6725	0.0625
Macronutrients							
Protein (g/kg BW/day)	1.33 (0.99 (1.67)	1.33 (1.00; 1.84)	1.48 (1.22; 1.79)	0.0055	0.1032	0.0025	0.3337
Carbohydrates (%E)	54.1 (49.8; 58.6)	56.9 (49.9; 60.7)	48.6 (44.6; 53.6)	0.0002	0.1148	0.0002	0.0002
Free sugars (%E) [4]	11.6 (8.1; 15.4)	6.4 (4.0; 9.0)	10.8 (7.7; 15.8)	0.0002	0.0002	0.8338	0.0002
Dietary fiber (g/1000 kcal)	14.7 (12.3; 17.6)	21.7 (18.2; 25.4)	11.7 (9.6; 13.7)	0.0002	0.0002	0.0002	0.0002
Fat (%E)	33.1 (29.0; 38.4)	39.9 (25.8; 37.0)	37.4 (31.4; 41.3)	0.0014	0.0186	0.0318	0.0004
SFA (%E)	13.1 (10.1; 15.8)	8.1 (5.9; 10.3)	16.1 (13.3; 19.2)	0.0002	0.0002	0.0002	0.0002
MUFA (%E)	10.6 (8.9; 12.9)	9.8 (7.8; 13.1)	12.3 (10.5; 14.1)	0.0175	0.2072	0.0291	0.0064
PUFA (%E)	6.5 (4.8; 8.3)	8.7 (7.2; 11.0)	5.1 (4.0; 5.9)	0.0002	0.0002	0.0004	0.0002
Vitamins							
Retinol-Equivalents (µg/1000 kcal)	447 (314; 631)	457 (320; 668)	438 (335; 611)	0.2670	0.7685	0.1423	0.1681
Tocopherol-Equivalents (mg /1000 kcal)	7491 (5777; 9527)	9667 (7918; 11517)	6012 (4864; 7477)	0.0002	0.0047	0.0004	0.0002
Vitamin C (mg/1000 kcal)	44 (31; 62)	64 (42; 83)	42 (28; 59)	0.0497	0.0158	0.6850	0.0933
Folate-Equivalents (µg/1000 kcal)	119 (103; 147)	150 (126; 178)	106 (82; 127)	0.0002	0.0047	0.0193	0.0002
Vitamin B1 (µg/1000 kcal)	440 (360; 525)	605 (496; 691)	455 (408; 534)	0.0002	0.0002	0.0747	0.0055
Vitamin B2 (µg/1000 kcal)	480 (381; 583)	373 (297; 455)	538 (453; 625)	0.0002	0.0002	0.0295	0.0002
Vitamin B12 (µg/1000 kcal)	0.6 (0.3; 1.1)	0.1 (0.0; 0.2)	1.6 (1.2; 2.0)	0.0002		0.0002	

Minerals								
Calcium	(mg/1000 kcal)	374 (281; 494)	302 (229;408)	401 (332; 474)	0.0035	0.0020	0.5885	0.0026
Magnesium	(mg/1000 kcal)	175 (153; 206)	251 (205; 304)	152 (134; 173)	0.0002	0.0002	0.0019	0.0002
Iron	(mg/1000 kcal)	6.7 (5.7; 7.8)	9.2 (7.5; 10.7)	5.7 (5.1; 6.3)	0.0002	0.0002	0.0018	0.0002
Zink	(mg/1000 kcal)	4.5 (3.9; 5.3)	5.1 (4.3; 6.0)	4.9 (4.4; 5.5)	0.0002	0.0002	0.0131	0.1465

Data are unadjusted median (Q1; Q3); BW Body weight, %E Percentage of energy intake

1 Eleven participants did not provide a dietary record, 72 records were classified as underreported

2 Analysis of covariance, adjusted for age (years), BMI-SDS, socioeconomic status (low/middle/high), smoking in the household (yes/no), physical activity (MET-minutes), use of dietary supplements (yes/no), *P*-values were adjusted for multiple testing according to the False Discovery Rate (FDR) method

3 excluding beverages

4 added sugars plus sugar from juices

SUPPLEMENTAL TABLE 2 Nutrient biomarker and blood lipids of children and adolescents of the German VeChi Youth Study (n = 365[1], 6-18 years old) stratified by diet group, excluding those participants consuming caloric food or drinks before blood withdrawal

	Vegetarian (n = 134)	Vegan (n = 102)	Omnivore (n = 129)	Total model	Pairwise comparison		
					VG-VN	VG-OM	VN-OM
	P50 (P25; P75)	P50 (P25; P75)	P50 (P25; P75)	P^2	P^2	P^2	P^2
Hemoglobin (g/dL)	13.3 (12.4; 14.2)	13.3 (12.6; 14.1)	13.5 (12.9; 14.2)	0.9102	0.9214	0.5612	0.7006
Ferritin (µg/L)	28 (20; 39)	29 (22; 42)	37 (26; 48)	0.0736	0.9214	0.0299	0.0954
25-OH-Vitamin D3 (µg/L)	24 (17; 31)	27 (20; 33)	24 (18; 31)	0.5633	0.8833	0.3230	0.5633
Vitamin B2 (FAD) (µg/L)	200 (177; 223)	196 (171; 216)	206 (190; 228)	0.2948	0.4159	0.3230	0.1516
Folate (µg/L)	280 (250; 320)	319 (287; 363)	282 (252; 327)	0.0248	0.0096	0.3677	0.1791
HoloTC (pmol/L)	57 (41; 84)	69 (44; 111)	67 (50; 83)	0.0514	0.5218	0.0205	0.2642
MMA (nmol/L)	153 (127; 195)	144 (110; 178)	153 (119; 195)	0.2426	0.9214	0.1223	0.2642
TC (mg/dL)	147 (130; 163)	134 (121; 153)	153 (133; 173)	0.0096	0.0205	0.2367	0.0048
HDL-C (mg/dL)	55 (47; 68)	53 (46; 64)	57 (48; 66)	0.8102	0.5633	0.8941	0.5633
Non-HDL-C (mg/dL)	89 (74; 100)	79 (65; 95)	96 (73; 113)	0.0067	0.0205	0.1516	0.0024
LDL-C (mg/dL)	80 (69; 93)	71 (58; 87)	90 (69; 106)	0.0032	0.0096	0.1570	0.0024
Triglyceride (mg/dL)	67 (54; 87)	69 (53; 84)	61 (51; 78)	0.3230	0.9214	0.1742	0.3230

Data are unadjusted median (Q1; Q3); holoTC Holo-Transcobalamin, MMA Methylmalonic acid, TC Total cholesterol

[1] Five participants did not provide a blood sample, further missings values resulted from insufficient sample volume or technical problems during analysis (folate n = 3, ferritin n = 1, vitamin B2 (FAD) n = 4, holoTC n = 7, hemoglobin and MMA n = 2), 31 samples were excluded after consuming caloric food or drinks before blood withdrawal

[2] Analysis of covariance, adjusted for age (years), BMI-SDS, socioeconomic status (low/middle/high), smoking in the household (yes/no), physical activity (MET-minutes), use of dietary supplements (yes/no), and season (only 25-OH-vitamin D3), P -values were adjusted for multiple testing according to the False Discovery Rate (FDR) method
